# How the natural environment affects psychological recovery: A case study in Changsha, China

**DOI:** 10.1371/journal.pone.0325755

**Published:** 2025-06-17

**Authors:** Kexin Zhao, Shuangquan Zhang, Fengying Zhao

**Affiliations:** 1 College of National Park and Tourism, Central South University of Forestry and Technology, Changsha, Hunan, China; 2 National Forestry and Grassland Engineering Research Center for Forest Tourism, Central South University of Forestry and Technology, Changsha, China; Shenyang Jianzhu University, CHINA

## Abstract

Previous research has demonstrated the role of urban forest parks in the recovery of human psychological well-being, but there is a lack of explanation of the “how” and “why” of experiences in urban forest parks that promote psychological recovery. This study used the structural equation model (SEM) method to explore the influence mechanism and path of the natural environment on the public’s psychological recovery evaluation in urban forest parks through 485 questionnaires. The study findings reveal the following: (1) In urban forest parks, natural environment perception (NEP) exerts significant positive effects on leisure involvement (LI), place attachment (PA), and restorative environment perception (REP). While NEP does not directly influence psychological recovery evaluation (PRE), its effects are mediated through four distinct pathways: First, via the complete mediation of REP; Second, through the serial mediation of LI and REP; Third, by the chain mediation of PA and REP; Fourth, through the sequential chain mediation of LI, PA, and REP. (2) Leisure involvement significantly enhances both place attachment and restorative environment perception. Additionally, place attachment exhibits a significant positive effect on restorative environment perception, while restorative environment perception demonstrates a robust positive association with psychological recovery evaluation. (3) Demographic variables, including gender, age, and educational level, did not demonstrate significant moderating effects on the structural relationship between natural environment perception and psychological recovery evaluation. The findings may have the potential to offer fresh ideas for enhancing the recovery effect of urban forest parks and have significant implications for their management and sustainable development.

## 1. Introduction

People are becoming more concerned about the potential restorative effects of the natural environment due to social problems caused by urban stress and the growing demand for health [1,2], and recovering physical and mental health has become the primary purpose of people’s trips [3]. Tourism destinations with natural landscapes exhibit significant restorative efficacy in environmental recovery. Urban forest parks are considered to be beneficial spiritual restoration landscapes in the urban environment [4], which have good ecological service functions and environmental restoration effects and have sound physiological and psychological recovery effects for the public [5,6]. These restorative soundscapes [7,8] and environmental characteristics [9] constitute a critical determinant of public visitation to urban forest parks.

Evidence is accumulating that natural landscapes have a stronger positive impact on health. Three aspects are largely responsible for this: short-term recovery from stress or mental fatigue, rapid recovery from disease [10], and long-term comprehensive improvement of people’s health and well-being [11]. In environmental psychology, the term “restoration” is an umbrella term that refers to the experience of a mental and physiological recovery process triggered by a particular environment and environmental configuration. An environment can be defined as a restorative environment when it exhibits characteristics such as “being away”, “fascination”, “extent”, and “compatibility”. The “restorative environment” is an environment that supports people in recovering and renewing from physical and mental exhaustion and negative emotions related to stress [12]. The psychological recovery and emotional restoration that individuals obtain through such environments are termed restorative experience or perceived restorativeness in environmental psychology literature.

The Stress Recovery Theory (SRT) [13] posits that psychological restoration arises from exposure to restorative environments that alleviate stress and reduce negative emotions. Empirical evidence suggests that natural environments are more conducive to improving physical and mental well-being compared to urban settings. Complementarily, the Attention Restoration Theory (ART) [14] emphasizes the critical role of directed attention in human information processing. Prolonged exertion of directed attention leads to cognitive fatigue, which negatively impacts daily functioning. To replenish attentional resources and repair perceptual deficits, individuals must engage with restorative environments, thereby mitigating directed attention fatigue through mechanisms such as fascination and psychological detachment. Empirical studies have delved into the effects of park landscape features, visitor activities, and service facilities on visitors’ psychological restoration, including but not limited to Li et al. (2023) [15] constructed PLS-SEM to quantitatively analyze the psychological recovery mechanism of urban forest environments. FEI et al.(2023) [16] analyzed how citizens felt about visiting forests using the Perceived Restorativeness Scale (PMS) and the Psychosocial Well-being Index Short Form (PWI-SF). The results show that the higher the perceived resilience is, the lower the psychosocial stress is.

While the restorative effects of natural environments have been widely studied, several research gaps remain. First, most studies have focused on general natural landscapes, with limited attention to the specific restorative mechanisms of urban forest parks [17]. Urban forest parks, as integrated natural spaces within urban settings, may offer unique restorative benefits due to their accessibility and the combination of natural and urban elements. However, the specific pathways through which these parks contribute to psychological restoration are not yet fully understood.

As demonstrated in the extant literature, place dependence affects all dimensions of restorative environmental perception, and place identity partially mediates this relationship, as evidenced by the results. Psychological recovery is influenced by place dependence through place identity, psychological escape, or infatuation. Furthermore, psychological recovery is influenced by place identity and can also have an indirect effect through infatuation. The dimensions of psychological escape and obsession in restorative environment perception also influence psychological recovery [15]. Despite the exploration of the relationships between perceived natural environment perception, leisure involvement, place attachment, restorative environment perception, and psychological recovery evaluation as separate entities, there is a paucity of integrated models that examine how these variables interact [18]. For instance, while place attachment has been demonstrated to influence perceived restorativeness, the mediating role of leisure involvement in this relationship remains under-explored [19].

Based on the current research status, this study aims to address the limitations of previous research by employing structural equation modeling to reveal the pathway mechanism of “natural environment perception (NEP)→leisure involvement (LI)/place attachment (PA)/restorative environment perception (REP) → psychological recovery evaluation (PRE)”. It systematically addresses the core questions of “how” and “why” urban forest park experiences promote psychological recovery, thereby deepening the explanatory framework of environmental psychology and restorative environment theory. Significantly, this research introduces leisure involvement and place attachment as mediating variables into the psychological recovery mechanism model, transcending the singular environmental cognitive perspective traditionally emphasized in Attention Restoration Theory (ART) and Stress Reduction Theory (SRT). This approach enriches the “cognitive-affective” model by integrating multidimensional perspectives. The findings further demonstrate that distinct environmental elements (e.g., vegetation morphology, spatial openness) indirectly influence psychological recovery effects through mediating variables. These insights suggest that urban planners should extend beyond ecological functionality to strategically design landscape features that stimulate recreational activities and enhance place identity, thereby optimizing the psychological restorative potential of urban green spaces.

## 2. Hypotheses and the conceptual model

### 2.1. The foundational driving role of natural environment perception

Environmental perception constitutes a fundamental form of human-nature interaction, serving as a mechanism through which individuals define themselves in relation to their surroundings. Positive human perceptions of natural environments, such as green spaces and water landscapes, can evoke pleasurable emotions, thereby enhancing the depth and continuity of recreational experiences. For instance, Cheng et al. (2014) [20] used SEM to examine how environmental perception affects behavior through recreational involvement.

It is well-documented that visiting or viewing a forest scene positively impacts psychological healing and well-being, particularly when recovering from stress [21]. Li et al. (2023) [15]demonstrated that, in the context of urban forests, natural environment perception directly influences residents’ psychological recovery evaluation and mediates the relationship between urban forest environments and psychological recovery. Similarly, Liu et al. (2021) [22] evaluated the relationship between restorative environment perceptions and their physiological and psychological impacts across different forest types, confirming a significant positive correlation between restorative environment perception and psychological recovery. The study conducted by Liu et al. (2018) [22] revealed that perceived nature and self-evaluation recovery (emotional, physiological, cognitive, and behavioral) had a significant correlation. Regarding the relationship between natural environment perception and restorative environment perception, Chen et al. (2023) [23] revealed that human recovery in forest environments is a progressive process, transitioning from spatial cognition to emotional responses. Their findings highlight that perceptions of the natural attributes and morphological features of recreational spaces play a pivotal role in the restorative effects of environments on individuals. Furthermore, a study examined the relationship between the perceived sensory dimension (PSD) of urban green space (UGS) and adolescents’ perceptions of recovery, stress, and mental health. The results showed that ‘nature’ was positively correlated with adolescents ‘perceived recovery (namely fascination, physically away, and extent), and ‘shelter’ was positively correlated with perceived recovery (namely being away from the body) [24]. Based on the above, the following hypotheses are proposed:

H1a: Natural environment perception has a significantly positive effect on leisure involvement

H1b: Natural environment perception has a significantly positive effect on restorative environment perception

H1c: Natural environment perception has a significantly positive effect on place attachment

Li et al. (2023)H1d: Natural environment perception has a significantly positive impact on the psychological restorative evaluation

H4: Restorative environmental perception has a significantly positive impact on psychological restorative evaluation

H6: Restorative environment perception mediates the effect of natural environment perception on psychological recovery evaluation

### 2.2. Interplay pathways of leisure involvement, place attachment, and restorative environment perception

Natural Environment Perception drives psychological recovery evaluation by stimulating profound leisure involvement, revealing a critical mediation pathway in the restorative environment mechanism. Research has demonstrated that the extent to which the public is involved has a direct impact on their engagement in tourism activities. When the public concentrates on enjoying the tourism process and deeply experiencing tourism activities, their place identity with the destination is enhanced, and their perception of the natural environment, social services, and tourism products of the destination is gradually deepened, there will also be a greater awareness of the importance of health recovery [25]。Numerous scholars have examined the connection between leisure involvement and place attachment, and leisure involvement was the antecedent variable for place attachment in an empirical study of different case sites. Wang et al. (2020) [26] took Fudao in Fuzhou City as an example to explore the relationship between leisure involvement, place attachment, and well-being. They verified that leisure involvement has a significant positive impact on place attachment. Tsung et al. [27], through their study on dog walkers in urban parks, confirmed that the higher the level of recreational activity participation among urban park visitors, the more their sense of place attachment tends to increase. Cheng et al. (2023) [28] divided recreation involvement into behavioral and psychological involvement and believed that the two dimensions of tourism involvement can positively affect the public’s restorative environment perception, and psychological involvement has the greatest impact on restorative environment perception. Yang et al. (2022) [29] observed that leisure involvement significantly affects how people perceive environmental restoration in ice and snow tourism scenes.A correlation has been demonstrated between place attachment and restorative environmental perception. The notion of a tourist’s favourite location being a site of profound attachment is a salient one. This is due to the fact that such places often provide a safe and comfortable environment that allows individuals to self-regulate, recover from stress, and focus on problem-solving and self-reflection. Menatti et al. (2019) [30] demonstrated that human-place connections significantly influence landscape preferences and the perception of their restorative attributes. Liu et al. (2020) [31] further identified that place dependence positively contributes to perceived restorativeness.

Based on the above, the following hypotheses are proposed

H2a: Leisure involvement has a significant positive effect on place attachment

H2b: Leisure involvement has a significant positive effect on restorative environmental perception

H3: Place attachment has a significantly positive effect on restorative environmental perception

H5: Leisure involvement and restorative environment perception sequentially mediate the effect of natural environment perception on psychological recovery evaluation

H7: Place attachment and restorative environment perception sequentially mediate the effect of natural environment perception on psychological recovery evaluation

H8: Leisure involvement, place attachment, and restorative environment perception sequentially mediate the effect of natural environment perception on psychological recovery evaluation

### 2.3. Moderating effects of demographic characteristics

Schipperijn et al. (2010) [32] employed nationwide Danish survey data to analyze the moderating roles of age, educational attainment, and income in determining the frequency and purposes of green space utilization. Complementing this, Bąkowska-Waldmann et al. (2023) [33] empirically demonstrated gender-based disparities in green space preferences and accessibility patterns: their findings reveal that female users tend to frequent green areas proximal to their residences and prioritize urban core green spaces, whereas male counterparts exhibit stronger preferences for remote green zones and demonstrate greater willingness to traverse extended distances for access. Meng’s (2008) [34] seminal work identified significant gender-based disparities in perceived importance of destination attributes and travel values when selecting nature-oriented tourism destinations. The findings reveal that female travelers prioritize appreciation of natural landscapes and recreational activities emphasizing relaxation, whereas their male counterparts demonstrate stronger preferences for challenging outdoor pursuits and adventure-driven experiences. Complementing this perspective, Jim et al. (2013) [35] investigated perceptual disparities in urban green space evaluations among Guangzhou residents, with particular emphasis on the moderating effects of income and educational attainment. Parallel methodological rigor characterizes Kabisch et al.’s (2014) [36] examination of socio-spatial equity in Berlin’s green space distribution, which systematically analyzed how income and education levels moderate usage patterns. This constellation of empirical evidence collectively substantiates the significant influence of demographic characteristics—including age, educational background, occupational status, and annual income—on urban forest park selection, destination attribute perception, and leisure experience quality. Consequently, visitors’ environmental perception demonstrates gender-mediated variations that subsequently shape psycho-environmental restoration outcomes. Therefore, this study posits the following hypotheses:

H9a: Gender differences significantly moderate the relationship between natural environment perception and psychological recovery evaluation

H9b: Age variations exert significant moderating effects on the association between natural environment perception and psychological recovery evaluation

H9c: Educational attainment demonstrates significant moderation in the linkage of natural environment perception with psychological recovery evaluation

H9d: Occupational status significantly influences the strength of the relationship between natural environment perception and psychological recovery evaluation

H9e: Annual income level serves as a significant moderator in the natural environment perception-psychological recovery evaluation pathway.

**Fig 1:**
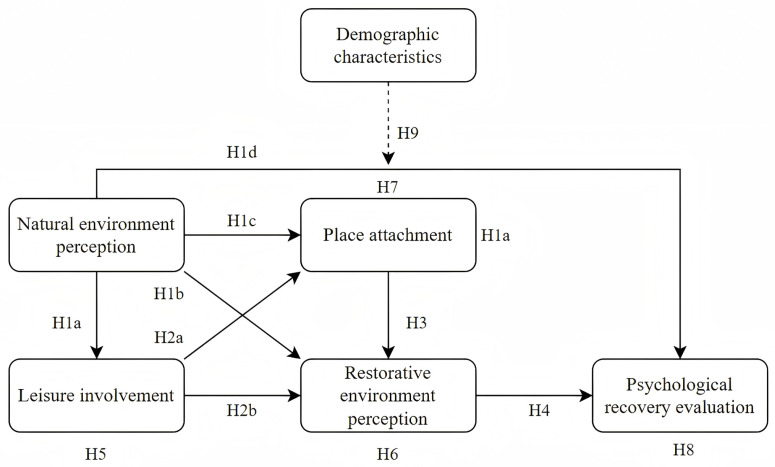
Research model.

Based on the literature review above, a hypothetical conceptual model diagram of the effects of psychological recovery evaluation mechanisms was constructed, as shown in Fig 1.

## 3. Methods

### 3.1. Study area

**Fig 2:**
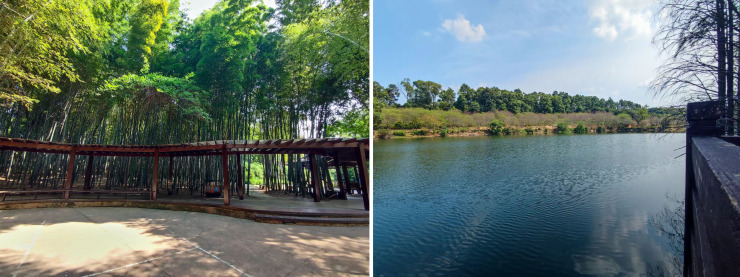
The Bamboo Garden and Mandarin Duck Lake within the Hunan Botanical Garden. (author’s own photo).

The Hunan Botanical Garden (Fig 2) is located in Changsha, the capital city of Hunan Province, China. It covers an area of 140 hectares, with a forest coverage of up to 90%. The garden has introduced, domesticated, relocated and preserved over 4,000 plant varieties from 208 families and 900 genera, representing more than 3,200 species. The garden is located in close proximity to the densely populated centre of Changsha, and is fully equipped with the leisure and recreation, and environmental restoration functions of urban forest parks. It is a typical representative of Changsha’s urban forest parks, and is now a national 4A level tourist attraction, developed for the public free of charge. It is one of the most important places for Changsha residents to have health, leisure, and recreation, and is an excellent research site for studying.

### 3.2. Questionnaire design

The Demographic Information section of the study recorded the gender, age, income, occupation, and education level of the participants. The measurement of natural environmental perception was mainly revised based on the research results of Kaltenborn et al. (2002) [37] and Zheng (2017) [38]. With a total of 6 questions, the course consisted of two dimensions: natural space perception and natural form perception. The measurement of leisure involvement is based on McIntyre et al.‘s (1992) EIS scale [39]. Twelve questions are included for three dimensions: attraction, centrality, and identity expression. The measurement of place attachment is based on William’s (2000) [40] scale. The questionnaire is divided into two dimensions, place dependence, and place identity, with 8 questions. Restorative environmental perception is measured based on the PDRQS scale developed by Lehto et al. (2013) [41]. The questionnaire is divided into four dimensions, compatibility, mental away, hysical away, and fascination, with 12 questions. The measurement of psychological recovery evaluation mainly refers to the research results of Kim et al. (2014) [42], Liu et al. (2018) [23], and others. The indicators are revised accordingly, and finally, the restoration evaluation scale in the Chinese context is formulated. It includes three dimensions: mental recovery, emotional recovery, and attention recovery, with a total of 12 questions (Table 1).

**Table 1:** Measurement items.

Variables	Variable quantity	Potential items	References
Natural environment perception	NEP1	It’s a quiet place	Kaltenborn et al. [37] Zheng [38]
	NEP2	The area is vast	
	NEP3	The landscape here is continuous	
	NEP4	The forest vegetation here is high and low, rich in layers	
	NEP5	The sounds of nature are varied and beautiful	
	NEP6	The natural environment of the area through which the road winds and flows is also varied	
Leisure involvement	LI1	It’s important for me to travel here	McIntyre et al. [39]
	LI2	Traveling here is one of the most satisfying things I’ve ever done	
	LI3	There are a lot of things in my life that are connected here	
	LI4	Visiting here is an important part of my life	
	LI5	If I want to change my preference for other activities, I have to rethink	
	LI6	When I play here, I can show my talent and personality	
	LI7	When a person is playing here, you can learn a lot about him	
	LI8	I love talking to my friends about the fun of being here	
	LI9	Most of my friends love to visit here	
	LI10	Visiting here is a great opportunity to spend time with friends	
	LI11	Here I can be my true self	
	LI12	When I play here, I can relate to the people and the sights	
Place attachment	PA1	This is more than other tourist destinations to meet my needs	William [40]
	PA2	The travel and leisure experience I have given here is irreplaceable by other tourist destinations	
	PA3	It’s the best place for me to do what I love	
	PA4	Compared to other urban forest park destinations, the experience of traveling here is more important to me	
	PA5	I feel like it’s a part of my life	
	PA6	This place is special to me	
	PA7	Visiting here helps me find myself	
	PA8	This place means a lot to me	
Restorative environment perception	REP1	I feel at ease here	Lehto et al. [41]
	REP2	Everything here is friendly	
	REP3	I felt at one with nature	
	REP4	Here, I can forget about responsibilities and stress for a while	
	REP5	Here, I felt a sense of seclusion and peace	
	REP6	Here I have a sense of being free from the shackles of the worldly world	
	REP7	Here, I feel that the environment I am in is different from the daily life environment	
	REP8	Here, what I do is not the same as what I do at home	
	REP9	The environment here is very different from what I am in on a daily basis	
	REP10	There are many interesting things to explore and discover	
	REP11	It’s full of charm	
	REP12	This is a place that I am very nostalgic for	
Psychological restorative evaluation	PRE1	This trip has boosted the motivation of my life	Kim et al [42], Liu et al. [43]
	PRE2	This trip has increased the value of my life	
	PRE3	This trip has boosted my self-confidence	
	PRE4	This trip has increased my sense of responsibility	
	PRE5	This trip has taken the stress off my shoulders	
	PRE6	The trip stabilized my mood	
	PRE7	I feel that life is full of fun	
	PRE8	This trip helped me develop a positive attitude towards life	
	PRE9	This trip helps me focus on one thing	
	PRE10	This trip helps me spend more time focusing on one thing	
	PRE11	This trip helped me mitigate the interference of external factors	
	PRE12	This trip helped me improve my work (study) efficiency	

### 3.3. Data collection

A random sampling survey was conducted on the public to Hunan Botanical Garden between September 28th and October 7th, 2023. The administration of the questionnaires was conducted in person by the research team. Potential respondents were distributed to the relevant locations by the research team, who also confirmed their willingness to participate and collected the completed questionnaires on-site. In order to align with the leisure activity patterns and preferences of visitors to Hunan Botanical Garden, the majority of data collection was conducted between 10:00 AM and 5:00 PM. Primary data collection sites included the walking paths adjacent to the Bamboo Garden (Zhuyuan) and Mandarin Duck Lake (Yuanyang Hu). The selection of these locations was driven by two primary factors. Firstly, these areas boast high-quality landscape resources that vividly exemplify the restorative environmental impacts of urban forest parks. Secondly, these sites are equipped with ample resting facilities and are situated along the main thoroughfares of the botanical garden, making them accessible to visitors and thus attracting a large and concentrated flow of leisure-seeking individuals. Upon distribution of the questionnaires, all participants provided oral consent and expressed their willingness to complete and submit the survey in its entirety. This survey constituted the consent form. For participants under the age of consent who were involved in the study, the oral consent of their legal guardians had been obtained. The anonymity of the participants was preserved throughout the study, and patients were at liberty to withdraw from participation at any time if they felt uncomfortable. Furthermore, each questionnaire was designed to be completed within 3 minutes to ensure respondents remained focused during the survey process. The questionnaire incorporated logic-check questions and repeated items to evaluate response consistency, thus facilitating the identification and exclusion of invalid responses, thereby enhancing the quality of the data. Following the completion of the survey, the collected data were promptly organised and analysed to detect and address any anomalies or outliers. A total of 513 questionnaires were distributed, and 457 valid samples were collected after the screening and collation, with an effective sample recovery rate of approximately 89.1%.

### 3.4. Data analysis

The analytical process comprised three sequential phases using SPSS, AMOS, and SPSSAU platforms. Initially, SPSS facilitated data screening and reliability validation, confirming internal consistency through Cronbach’s α coefficients exceeding 0.70. Subsequently, AMOS-based confirmatory factor analysis (CFA) established measurement model validity, demonstrating strong scale stability with optimized fit indices (CFI = 0.93, RMSEA = 0.05). Finally, structural equation modeling (SEM) implemented via SPSSAU elucidated pathway relationships and mediation effects, specifically investigating the mechanisms through which urban forest environments facilitate psychological restoration. This integrated analytical framework ensured both psychometric rigor and theoretically grounded exploration of environmental restoration dynamics.

## 4. Results

### 4.1. Demographics

The population distribution of the respondents is shown in Table 2. Of the 457 respondents, 49.9% were male, and 50.1% were female. 60.8% of the total respondents were aged 18–34 years old when it came to age. Students, employees/workers, and Specialized technical staff accounted for 66.9% of the total respondents. About 84.9% of the respondents had a college or university degree or above. 52.5% of the respondents had an average annual household income of more than 24,000 yuan.

**Table 2:** Descriptive statistical results.

Indicator	Item	Frequency	%
Gender	Female	229	50.1%
	Male	228	49.9%
Age	Under 18	27	5.9%
	18–24	178	38.9%
	25–34	100	21.9%
	35–44	62	13.6%
	45–54	51	11.2%
	55–64	30	6.6%
	65 above	9	2%
Education	Junior Secondary and below	20	4.4%
	High School and Secondary School	49	10.7%
	College and Undergraduate	314	68.7%
	Postgraduate and above	74	16.2%
Personal annual income	Under 24000	217	47.5%
	24000–60000	55	12.0%
	60001–120000	106	23.2%
	120000 above	79	17.3%
Job	Civil services	27	5.9%
	Managers of enterprises and institutions	47	10.3%
	Employees/workers	57	12.5%
	Private owners	19	4.2%
	Military	2	0.4%
	Unemployed/laid-off	3	0.7%
	Specialized technical staff	61	13.3%
	Peasants	4	0.9%
	Students	188	41.1%
	Retired	14	3.1%
	Other	35	7.7%

### 4.2. Common method variance test

To mitigate potential analytical biases arising from the fact that all questionnaire items were derived from a single instrument and consistently reported by the same respondents, and to ensure compliance with standardized pre-analytical protocols for quantitative survey data, Harman’s single-factor test was initially employed to assess common method bias (CMB) across the sample. Specifically, an unrotated exploratory factor analysis was conducted on all measured variables. The dataset was considered free from significant common method variance if no single factor accounted for the majority of the variance (>50%), as per established psychometric thresholds [44]. The first principal component explained 35.213% of the variance, less than half of the total variance (75%). The study does not have a significant common method bias.

### 4.3. Reliability and validity analysis of structural equation models

The collected questionnaire data were loaded into SPSS27.0 for reliability and validity analysis. As shown in Table 3 below, the overall reliability of the scale is 0.939, and the reliability of each variable is also above 0.8, indicating that the reliability of the scale is good; the KMO of the scale is 0.957, and the KMO of each variable also meets the basic requirement of 0.8 and above; Bartlett’s test of sphericity also achieves the level of significance, which indicates the validity of the data for factor analysis.

**Table 3:** Reliability and validity tests.

Item	Cronbach’s Alpha	Kaiser-Meyer-Olkin	Bartlett’s Test of Spherical	Sig.
Overall table	0.939	0.957	14936.73	0
Natural environment perception	0.826	0.855	866.094	<0.001
Leisure involvement	0.911	0.924	2716.649	0
Place attachment	0.904	0.916	1908.465	0
Restorative environment perception	0.912	0.911	2829.085	0
Psychological recovery evaluation	0.928	0.919	3506.508	0

Table 4 shows how the sample data was subjected to a validation factor analysis using the maximum likelihood estimation method using Amos 28.0 software. All variables were found to have composite reliability (CR) above 0.7. The average variance extracted (AVE) of respective items was 0.449, 0.466, 0.533, 0.439, and 0.490. According to Fornell and Larcker (1981) [45] standards, CR should exceed 0.6, and AVE should exceed 0.5 under ideal condition, while 0.36 ~ 0.5 are acceptable. Hence, all items for convergent validity were met, which further validating the internal consistency and stability of the variables measured in the questionnaire.

**Table 4:** Results of the validation factor analysis.

Factor	Dimensions	Items	Estimate	AVE	CR
Natural environment perception	Natural space perception	NSP1	0.592	0.449	0.829
		NSP2	0.752		
		NSP3	0.707		
	Natural form perception	NFP1	0.708		
		NFP2	0.609		
		NFP3	0.637		
Leisure involvement	Attraction	ATT1	0.681	0.466	0.912
		ATT2	0.679		
		ATT3	0.7		
		ATT4	0.76		
	Centrality	CEN1	0.707		
		CEN2	0.882		
		CEN3	0.644		
		CEN4	0.621		
	Identity expression	IE1	0.647		
		IE2	0.581		
		IE3	0.63		
		IE4	0.604		
Place attachment	Place dependence	PD1	0.71	0.533	0.901
		PD2	0.684		
		PD3	0.725		
		PD4	0.72		
	Place identity	PI1	0.742		
		PI2	0.727		
		PI3	0.733		
		PI4	0.793		
Restorative environment perception	Compatibility	COM1	0.658	0.439	0.903
		COM2	0.7		
		COM3	0.657		
	Mentally away	SA1	0.66 2		
		SA2	0.662		
		SA3	0.645		
	Physically away	BA1	0.589		
		BA2	0.547		
		BA3	0.707		
	Fascination	ASC1	0.72		
		ASC2	0.68		
		ASC3	0.698		
Psychological recovery evaluation	Mental recovery	MR1	0.705	0.49	0.92
		MR2	0.708		
		MR3	0.732		
		MR4	0.705		
	Emotional recovery	ER1	0.655		
		ER2	0.693		
		ER3	0.73		
		ER4	0.755		
	Attention restoration	AR1	0.669		
		AR2	0.698		
		AR3	0.676		
		AR4	0.658		

To ensure questionnaire content validity, each question was taken from well-established scales in the literature in the questionnaire design. The variance contribution of the first principal component of each latent variable usually determines construct validity. The results showed that the contribution of the first principal component of natural environment perception, leisure involvement, place attachment, environmental restorative perception, and psychological recovery evaluation was 57.34%, 56.82%, 64.21%, 53.33%, and 54.80%, respectively. Generally, the requirement that the first principal component is greater than 40% is acceptable [46]. The structural validity of the volume scale is improved because the questionnaire scale’s measurement items contribute more to the corresponding latent variables. To further examine the discriminant validity, as shown in Table 5, there is a certain degree of correlation among the latent variables, while they also exhibit distinct separation from one another, indicating that the discriminant validity of the scale data is satisfactory. SEM was utilized to verify further.

**Table 5:** Discriminant validity analysis.

Variables	Natural environment perception	Leisure involvement	Place attachment	Restorative environment perception	Psychological recovery evaluation
Natural environment perception	**0.670**				
Leisure involvement	0.426	**0.683**			
Place attachment	0.434	0.658	**0.730**		
Restorative environment perception	0.533	0.662	0.710	**0.663**	
Psychological recovery evaluation	0.479	0.546	0.617	0.589	**0.700**

### 4.4. Structural equation model analysis

#### 4.4.1. Structural equation model fitting.

The structural equation model was subjected to rigorous scrutiny to ascertain its suitability, employing a range of fit indices, such as X2/df, CFI, and RMSEA [47]. The model’s performance was found to be within the acceptable limits, as outlined by Su et al. (2023) [48], thereby affirming its adequacy for the subsequent analysis. The outcomes of this investigation are delineated in Table 6. The model fitness effect at Hunan Botanical Garden has been found to be more satisfactory, with a high quality that renders it ideal for the next analysis step.

**Table 6:** Test of the degree of fit of the structural equation model.

Parameters	Reasonable Standard	Excellent Standard	Model Value	Judgment of parameters	Compliance or non-compliance
X^2/^df	<5	<3	2.064	Excellent	Compliance
GFI	>0.8	>0.9	0.831	Reasonable	Compliance
AGFI	>0.8	>0.9	0.806	Reasonable	Compliance
NFI	>0.8	>0.9	0.852	Reasonable	Compliance
CFI	>0.8	>0.9	0.917	Excellent	Compliance
RMSEA	<0.08	<0.05	0.048	Excellent	Compliance

#### 4.4.2. Path analysis.

**Fig 3:**
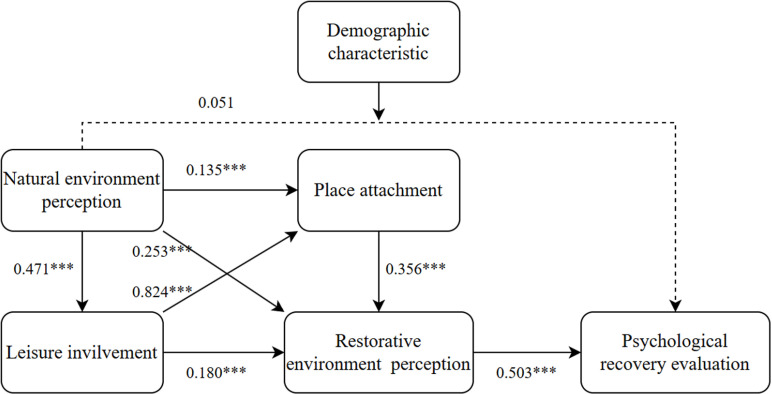
Results of path analysis.

Linear regression was employed to establish model relationships between one or more independent variables and the dependent variable. In this study, linear regression analysis was applied to investigate the pathway relationships within the structural equation model. The results demonstrate the following: Natural environment perception exerts significant positive effects on leisure involvement (estimate = 0.471, t = 10.042, p < 0.01), place attachment (estimate = 0.135, t = 3.680, p < 0.01), and restorative environment perception (estimate = 0.253, t = 7.541, p < 0.01), thereby confirming hypotheses H1a, H1b, and H1c. Leisure involvement significantly enhances place attachment (estimate = 0.824, t = 24.792, p < 0.01) and restorative environment perception (estimate = 0.180, t = 3.930, p < 0.01), supporting hypotheses H2a and H2b. Place attachment positively predicts restorative environment perception (estimate = 0.356, t = 8.434, p < 0.01), validating hypothesis H3. Restorative environment perception exhibits a significant positive influence on psychological recovery evaluation (estimate = 0.503, t = 11.525, p < 0.01), confirming hypothesis H4. However, no statistically significant relationship was observed between natural environment perception and psychological recovery evaluation (estimate = 0.051, t = 1.538, p = 0.125), leading to the rejection of hypothesis H1d. The pathway analysis results of this structural equation model are comprehensively summarized in Fig 3.

#### 4.4.3. Mediation analysis.

The mediation within the model was verified by performing Bootstrap sampling tests on the indirect effect values, and the presence of mediation effects was confirmed through Bootstrap analysis [49]. The interpretation of the results is based on whether 0 is included in the 95% confidence interval. If 0 is not, the study pathway is significant, but vice versa, it is not significant. Table 7 indicates that this structural equation model’s mediating effects include parallel and chained mediation. For the mediation pathway “Natural Environment Perception (NEP) → Restorative Environment Perception (REP) → Psychological Recovery Evaluation (PRE)”, the 95% confidence interval (CI) does not include zero (95% CI: 0.072–0.146), indicating the presence of a significant mediation effect. Thus, hypothesis H6 is supported. Regarding the mediation pathway “Natural Environment Perception (NEP) → Leisure Involvement (LI) → Restorative Environment Perception (REP) → Psychological Recovery Evaluation (PRE)”, the 95% confidence interval (CI) excludes zero (95% CI: 0.017–0.065), confirming the existence of this mediation effect. Consequently, hypothesis H5 is validated. For the mediation pathway “Natural Environment Perception (NEP) → Place Attachment (PA) → Restorative Environment Perception (REP) → Psychological Recovery Evaluation (PRE)”, the 95% confidence interval (CI) does not encompass zero (95% CI: 0.009–0.039), demonstrating a significant mediation effect. Therefore, hypothesis H7 is substantiated. Finally, for the mediation pathway “Natural Environment Perception (NEP) → Leisure Involvement (LI) → Place Attachment (PA) → Restorative Environment Perception (REP) → Psychological Recovery Evaluation (PRE)”, the 95% confidence interval (CI) excludes zero (95% CI: 0.039–0.094), providing evidence for the presence of this mediation effect. As a result, hypothesis H8 is confirmed.

**Table 7:** Results of indirect effects analysis.

Path	Effect	Boot SE	BootLLCI	BootULCI	*z*	*p*
NEP→REP→PRE	0.127	0.021	0.079	0.160	6.137	0.000
NEP→LI→REP→PRE	0.043	0.012	0.017	0.065	3.571	0.000
NEP→PA→REP→PRE	0.024	0.008	0.009	0.039	3.147	0.002
NEP→LI→PA→REP→PRE	0.069	0.014	0.039	0.094	4.926	0.000

Note: BootLLCI refers to the lower limit of the 95% confidence interval in Bootstrap sampling, while BootULCI denotes the upper limit of the 95% confidence interval in Bootstrap sampling. The bootstrap method employed is the percentile bootstrap approach.

#### 4.4.4. Moderating effect.

The moderating effects of gender, age, educational background, personal income, and occupation on the “nature-environment-psychological restoration” relationship were examined through hierarchical regression models. As shown in Table 8, none of the interaction terms in any model achieved statistical significance (p > 0.05), indicating the absence of significant moderating effects from demographic variables. It demonstrates that the standardized coefficient of natural environment perception (NEP → PRE) remained stable (β = 0.479–0.491) and statistically significant (p < 0.001) after incorporating moderators, suggesting the cross-group generalizability of nature’s psychological restoration effects. Consequently, Hypothesis H9 was rejected, confirming that demographic characteristics (gender/age/education/income/occupation) exhibit no significant moderating role in the “nature-environment-psychological restoration” pathway.

**Table 8:** Results of the moderating effect analysis.

Moderator	Interaction Term Coefficient (B)	p-value	ΔR²
Gender	0.000	0.999	0.000
Age	0.001	0.982	0.000
Educational background	−0.074	0.283	0.002
Personal income	0.007	0.848	0.000
Occupation	−0.018	0.179	0.003

## 5. Discussion

Not only does the research on the evaluation mechanism of the public’s psychological recovery in urban forest parks conform to the need for sustainable development of urban forest parks, but it also meets the needs of individual mental health development. Guided by environmental restoration theory, this study constructed a structural equation model to investigate the relationship between natural environment perception and psychological recovery evaluation. The proposed model established 13 hypothesized pathways, of which 12 were empirically supported based on the analytical results.

The study found that natural environment perception does not directly influence psychological restorative evaluation. Mediation is required through restorative environmental perception, leisure involvement, place attachment, and restorative environmental perception. This ultimately influences the effectiveness of psychological recovery evaluation, but it contradicts hypothesis H1d. This finding contradicts the study by Li et al. [15] but aligns with the research conducted by Chen et al. [23]. The primary discrepancy between these two studies likely stems from the differences in the scales adopted within their questionnaire designs for measuring natural environment perception. Li et al. utilized the Perceived Sensory Dimensions (PSD) scale, which dissects specific sensory attributes (e.g., “serenity,” “vitality,” and “shelter”) to unravel the distinct mechanisms of environmental features. In contrast, Chen et al. employed the Perceived Nature Scale (PNS) to evaluate individuals’ holistic perception of natural environments. While the PSD scale emphasizes fragmented sensory characteristics, the PNS scale prioritizes integrated environmental perception and emotional bonding with nature. Consistent with Chen et al., our study adheres to the environmental psychology perspective, positing that psychological recovery derived from natural environments is a multi-stage perceptual process, wherein individuals progressively engage with environmental stimuli through sequential cognitive and affective evaluations. Environmental psychology asserts that environmental perception is a process that is made up of three main elements: perception, cognition, and appraisal, all of which are connected [50]. The individual’s current needs and environmental characteristics play a role in influencing this. Senses of environmental stimuli are organized into higher-level mental models by the individual using existing experience from the perspective of environmental perception.

This study examined four mediation pathways through which natural environment perception influences psychological recovery evaluation, all of which demonstrated statistically significant mediation effects. These findings align with prior research outcomes [23], indicating that in urban forest parks, psychological factors such as emotional experiences, leisure involvement, and place attachment play critical roles in the psychological recovery process, in addition to the inherent restorative properties of natural environments. Visitors with higher levels of leisure involvement are more likely to find psychological solace in natural settings, thereby fostering a profound attachment to the park’s locality. This sense of place attachment not only deepens their environmental identity but also facilitates more pronounced psychological recovery effects.This finding corroborates the perspective of Korpela et al. (2001) [51], which posits that individuals tend to select environments congruent with their self-identity for stress regulation. For instance, high-involvement activities such as hiking or birdwatching not only enhance environmental immersion [52] but also establish “place memory” [53] through repetitive behaviors, thereby forming a positive feedback loop. Furthermore, this research integrates leisure involvement theory, place attachment theory, and restorative environment theory to investigate the restorative efficacy of urban forest parks. By exploring the psychological recovery evaluation mechanisms from the subjective perspective of visitors, it elucidates the synergistic interplay of multiple factors in the nature-psychological recovery nexus. The results validate the roles and theoretical positions of leisure involvement and place attachment theories while expanding their applicability to urban forest park contexts.

The study reveals that demographic variables, including gender, age, education level, occupation, and annual income, do not moderate the relationship between natural environment perception and psychological recovery evaluation. This finding contrasts with previous research [32,35]. Several factors may explain this discrepancy: First, it may stem from the homogeneity in public green space usage behaviors among urban residents in China. As a free-access urban forest park, Changsha Forest Park attracts a diverse range of visitors for daily recreation, potentially diminishing group-based differences [54]. Second, the collectivist orientation inherent in Confucian culture, particularly its social norms that prescribe communal utilization of natural spaces, could diminish the moderating role of individual traits in shaping their individual preferences [55]. This finding suggests that environmental psychology models centered on individual differences should be applied cautiously in non-Western contexts. Third, natural environment perception may represent a universal human experience, with shared perceptual responses to natural settings potentially transcending the influence of individual differences such as gender or age [56]. Fourth, prior research indicates that the influence of gender on environmental concern is not independent but mediated by environmental knowledge levels [57]. This implies that sociodemographic variables like gender may indirectly influence the NEP-PRE relationship through other psychological or behavioral factors. Thus, when these indirect pathways are not thoroughly examined, direct effects may appear non-significant.

The suitability and effectiveness of restorative environments have been the focus of more research results in environmental psychology research. Most employ quantitative and experimental methods to assess the quality of restorative environments, including photo ratings, eye tracking, choice-based conjoint analysis methods [58], and virtual reality [59]. Studies on restoring visitors to urban forest parks in the related field of urban forest parks are scarce. More extensive examinations of the pathways of the psychological evaluation mechanism still need to be done. Most studies on restorative environment perception in China utilize measurements that rely on the Kaplan(1995) [60] Perceptual Restoration Scale (PRS)‘s four dimensions of compatibility, fascination, remoteness, and extent [61]. This type of scale is only useful for evaluating the impact of environmental restoration at the cognitive level of restorative environment perception. Assessing whether the restorative effect of the destination environment is effectively realized requires considering the psychological and emotional recovery obtained through restorative environments. The psychological recovery evaluation scales introduced from abroad may also be inapplicable in the Chinese context, creating an urgent need for more localized studies to adapt the measurements to the Chinese public. Subiza-Pérez (2021) [62] used attention recovery, emotional recovery, and aesthetic experience to assess the participants’ environmental experience. The study site chosen was a city square that was part of an urban open space, with fewer green elements and biodiversity compared to botanical gardens, and the natural characteristics were not distinctive enough to fully fit the study’s research objectives. Based on previous studies, the study innovated the research perspective by taking individuals’ subjective evaluation of the impact of the natural environment on their physical and mental health as an important basis for evaluating whether the restorative effect of the destination’s environment is effectively exerted. A psychological recovery evaluation scale that applies to Chinese people in the Chinese context has been adopted. The tourist’s restorative environment experience is evaluated by determining five factors: mental recovery, emotional recovery, social recovery, physical recovery, and attention recovery. It made up for the shortcomings of the relevant foreign scales used in the Chinese context. It provided a new evaluation method for the research on the effect of a restorative environment on the public. The well-fitting between the model of the study and the sample data indicates the applicability and validity of the model in measuring and evaluating whether the restorative effects of urban forest park environments are effectively exerted. The restorative effectiveness of the environment can be assessed more reliably with these findings as a basis for further research. This indicates that the application of the study of theory and practice is of great significance and, to a certain extent, can guide improving the environmental restorative effectiveness of urban forest parks.

The present study still has some limitations. Firstly, although the evaluation mechanism for psychological recovery was examined during the study, the structural equation model utilized in the research only validated the relationship between the variables and did not investigate the elements contained within them. Individual elements were not deemed to impact the relationship between variables. Based on previous studies, conducting a more thorough analysis of the pathways that lead to the formation of psychological recovery evaluation mechanisms is necessary. To fully understand their interaction, it’s necessary to examine the interactions of elements in the variables, despite the current SEM providing the connection between them as a whole. Incorporating spatial characteristics and environmental components is crucial to elevating the standard of urban green space restoration [63]. Secondly, the survey was conducted during the period of September 21 to October 31, 2023, coinciding with the launch of the 2023 Hunan World Famous Flowers Eco-Cultural Festival “Autumn Colors” Flower Exhibition and the National Science Popularization Day event at Hunan Botanical Garden. During this period, multiple student groups participated in scientific, technological, and science popularization education activities organized at the site. Additionally, student participants exhibited a lower refusal rate compared to other demographic groups, consequently resulting in their overrepresentation in the survey sample. Future research should employ more balanced sampling strategies to ensure adequate representation of participants across diverse sociodemographic backgrounds and to rigorously examine potential variations among subgroups.

## 6. Conclusions

Building upon previous research, this study provides an in-depth exploration of the “how” and “why” behind the psychological restoration facilitated by urban forest park experiences. Based on the research findings, the main conclusions are as follows: (1) In urban forest parks, natural environment perception demonstrates significant positive effects on leisure involvement, place attachment, and restorative environment perception. Although natural environment perception does not directly influence psychological recovery evaluation, it exerts indirect effects through four mediating pathways: firstly, through the complete mediating role of restorative environment perception; secondly, via the serial mediation of leisure involvement and restorative environment perception; thirdly, through the chain mediation of place attachment and restorative environment perception; and fourthly, by means of the sequential mediation of leisure involvement, place attachment, and restorative environment perception. (2) Leisure involvement exhibits significant positive effects on both place attachment and restorative environment perception. Place attachment significantly enhances restorative environment perception, while restorative environment perception shows a significant positive impact on psychological recovery evaluation. (3) Demographic characteristics including gender, age, and educational level do not demonstrate moderating effects on the structural relationships between natural environment perception and psychological recovery evaluation. Collectively, this research elucidates that natural environment perception facilitates environmental intervention effects through a chain-mediated pathway involving leisure participation (behavioral dimension), place attachment (affective dimension), and perceived restorative environment (cognitive dimension). The findings advance theoretical frameworks by proposing novel structural equation models for Stress Recovery Theory (SRT) and Attention Restoration Theory (ART). These empirically validated mechanisms carry practical implications for urban forest park planning and management, enabling decision-makers to strategically design restorative landscapes that optimize the environmental rehabilitation potential of urban green spaces, thereby enhancing public psychological well-being and mental health.

## Supporting information

S1 Dataset The minimum data set. (XLSX)
